# Targeting the androgen receptor to enhance NK cell killing efficacy in bladder cancer by modulating ADAR2/circ_0001005/PD-L1 signaling

**DOI:** 10.1038/s41417-022-00506-w

**Published:** 2022-08-01

**Authors:** Qing Liu, Bosen You, Jialin Meng, Chi-Ping Huang, Guanglu Dong, Ronghao Wang, Fuju Chou, Shan Gao, Chawnshang Chang, Shuyuan Yeh, Wanhai Xu

**Affiliations:** 1 Department of Radiation Oncology, Urology, and Pathology, The Second Affiliated Hospital of Harbin Medical University, Harbin, 150001 China; 2 Departments of Urology, Pathology, Radiation Oncology, and The Wilmot Cancer Institute, University of Rochester Medical Center, Rochester, NY 14642 USA; 3 Department of Urology, China Medical University/Hospital, Taichung, 404 Taiwan; 4 Department of Urology, The 4th Affiliated Hospital of Harbin Medical University, Harbin, 150001 China

**Keywords:** Bladder cancer, Tumour immunology

## Abstract

Although androgen receptor (AR) can influence bladder cancer (BCa) initiation and progression, its impact on tumor immune escape remains unclear. Here, we found that targeting AR could enhance natural killer (NK) cell tumor-killing efficacy by decreasing PD-L1 expression. Both antiandrogen treatment and AR knockdown effectively reduced membrane PD-LI expression to facilitate NK cell-mediated BCa cell killing by downregulating circ_0001005. Mechanistically, AR upregulated circRNA circ_0001005 expression via the RNA-editing gene ADAR2. circ_0001005 competitively sponged the miRNA miR-200a-3p to promote PD-L1 expression. A preclinical BCa xenograft mouse model further confirmed this newly identified signaling using the small molecule circ_0001005-shRNA to improve NK cell killing of BCa tumor cells. Collectively, these results suggest that targeting the newly identified ADAR2/circ_0001005/miR-200a-3p/PD-L1 pathway to impact antitumor immunity may suppress progression and boost immunotherapeutic efficacy in BCa.

## Introduction

According to global cancer statistics from 2020, urinary bladder cancer (BCa) ranked as the 6th highest type of estimated new cancer cases among the ten leading cancer types in men, and it was the 9th leading cause of cancer-related death among men. The global incidence and mortality among men are approximately 4 times greater than those among women [1]. With the conspicuous morbidity and mortality in males, sex differences may play a critical role in BCa development [2]. Previous studies have revealed the role of the androgen receptor (AR) in the initiation and progression of BCa [3, 4]. Targeting the AR signaling pathway can affect chemosensitivity and Bacillus Calmette-Guerin (BCG) therapeutic efficacy [5, 6].

Programmed death ligand-1 (PD-L1) is an important immune checkpoint molecule highly expressed in tumor tissues that transduces immunosuppressive signals to suppress the antitumor efficacy of immune cells through PD-L1 binding to programmed cell death protein-1 (PD-1) on activated T cells [7, 8]. PD-1 is also expressed in natural killer (NK) cells according to published studies and might be involved in NK cell dysfunction in some tumors and correlate with a poor prognosis [9, 10]. PD-L1 expression in BCa is associated with higher grades/stages, tumor progression, a lack of responsiveness to BCG immunotherapy, and poor survival [11, 12]. Anti-PD-1/PD-L1 agents have been approved by the US Food and Drug Administration for the treatment of unresectable and metastatic urinary BCa. Patients with high PD-L1 expression tend to have shorter median overall survival than subsets of patients with low expression (12.3 vs. 19.1 months) given atezolizumab [13]. As combination strategies using immunotherapy together with targeted agents, chemo/radiotherapy, or other immune agents increasingly emerge, the potential to overcome the current issues of immunotherapies, such as partial responses to immune checkpoint therapy and immune checkpoint inhibition escape, may be realized, ultimately improving the overall survival of patients with urothelial carcinoma [14]. Likewise, tumors with relatively high expression of PD-1/PD-L1 have been indicated to have worse survival outcomes, which prompted the clinical use of PD-L1-targeted therapies.

As noncoding RNAs in cancer are receiving mounting attention due to their ex-tensive but unclear roles, studies on their functions involved in immune regulation are appearing [15, 16]. Growing evidence highlights functional roles for circular RNAs (circRNAs) in biological processes, including sponging microRNAs (miRNAs, miRs), regulating transcription, and modifying parental gene expression [17, 18]. With the detection of increasing numbers of circRNAs in many different types of cancers, circRNAs have been proven to play tumor-promotive or tumor-suppressive roles in cancer initiation and progression [17, 19–21]. In BCa, some recent studies have implicated circRNAs in the regulation of tumor proliferation, angiogenesis, invasion, and migration, impacting BCa development [22–25]. Nevertheless, there are few studies on the potential role of circRNAs in regulating PD-L1 expression.

We applied the online tool TRGAted to analyze BCa survival based on a protein dataset from The Cancer Genome Atlas (TCGA), and the results showed that PD-L1 expression had a significantly negative correlation with survival in male patients diagnosed with BCa but not in female patients. We expect that the expression of AR and PD-L1 may be significantly correlated with progression-free survival in different sub-types of BCa. Earlier studies have shown the significant roles of AR signaling in bladder tumorigenesis [26] and the promising suppressive efficacy of anti-androgen therapies [5]. In this paper, we demonstrate that knocking down AR expression and/or administering antiandrogens can substantially decrease PD-L1 expression in BCa cells, consequently enhancing the efficacy of NK cell killing of tumor cells. Targeting the AR signaling pathway may impact or remodel the tumor immune microenvironment to prevent tumor cells from escaping immunosurveillance, better suppress tumor progression and improve patient survival.

## Materials and methods

### Cell lines

The T24 and TCC-SUP BCa cell lines and NK-92 cell line were purchased from the American Type Culture Collection (ATCC, Manassas, VA). T24 and TCC-SUP cells were cultured in Dulbecco’s modified Eagle’s medium (DMEM) supplemented with 10% fetal bovine serum (FBS), and NK cells were cultured in α-MEM supplemented with 12.5% horse serum and 200 IU/mL hIL-2 (STEMCELL, MA, USA); both media were supplemented with antibiotics (100 units/ml penicillin and 100 mg/ml streptomycin), and 2 mM glutamine (Invitrogen, Grand Island, NY, USA). All cell lines were maintained in a humidified 5% CO2 environment at 37 °C.

### Lentivirus packaging and transfection

The plasmids pLKO.1-shAR, pLKO.1-shPD-L1, pLKO.1-shcirc_0001005 (cir-cASB3_016), pLKO.1-shcirc_0000005, pLKO.1-shcirc_0000713, pLKO.1-miR-200a-3p, pLKO.1-miR-219b-3p, pLKO.1-shADAR2-1# and 2#, pWPI-AR, and pWPI-circ ASB3_016 were co-transfected with the packaging and envelope plasmids psPAX2 and pMD2.G into HEK293T cells for 48 h following the standard CaCl2 transfection method to produce lentiviral particles suspended in the medium, which was filtered and then used immediately or stored at −80 °C for later use. circRNA overexpression plasmids were generated according to the method reported in our previous paper [27]. For cloning and PCR amplification of the circRNA from pBSK_circ_0001005, the forward primer was 5’-GTG AGG AAT TTC GAC ATT TAA ATT TAA TTC ATC TGA AAA CTA CAT TAA G-3’, and the reverse primer was 5’-TCC TGC AGC CCG TAG TTT CAA TAT GTT GCT GTA GAA TCC-3’.

### Reagents and materials

Antibodies against AR (N-20) and GAPDH (6c5) were purchased from Santa Cruz Biotechnology (Dallas, TX, USA). An anti-PD-L1 antibody was purchased from Cell Signaling Technology (Danvers, MA, USA). Anti-mouse/rabbit secondary antibodies for western blotting were purchased from Invitrogen. Enzalutamide (ENZA) was obtained from MedChemExpress (South Brunswick, NJ, USA) and applied at 10 μM. 5α-Dihydrotestosterone (DHT) from Sigma (St Louis, MO) was used at 10 nM. Hydroxyflutamide (HF) from AstraZeneca (Wilmington, DE, USA) was applied at 10 μM, and the AR-degradation enhancer ASC-J9^®^ was obtained from AndroScience Corp. (Solana Beach, CA, USA) and used at 5 μM. DHT, Enzalutamide and other anti-androgens (HF and ASC-J9) treatments were added in the cultured cells for 48 h to perform further experiments.

### Western blot

Cells were lysed in cell lysis buffer, and 30–50 μg of protein was mixed and boiled for equal loading, separated on 6–10% SDS/PAGE gels, and then transferred to PVDF membranes (Millipore, Billerica, MA, USA). We dissolved 5% skim milk in a TBST solution for blocking. After 1 h of blocking, the membranes were incubated with specific primary antibodies overnight, and then HRP-conjugated secondary antibodies were added for 1 h. An ECL system (Thermo Fisher Scientific, Rochester, NY, USA) was used for visualization. The primary antibodies used in the study included the following, anti-AR (sc-816, Santa Cruz, Dallas, TX, USA), anti-PD-L1 (#13684, Cell Signaling Technology, Danvers, Mass, USA), anti-GAPDH (sc-47724, Santa Cruz), anti-α-tubulin (sc-8035, Santa Cruz).

### RNA extraction and quantitative real-time PCR (qRT-PCR) analysis

Total RNA was extracted using TRIzol reagent (Invitrogen). Superscript III transcriptase (Invitrogen) was applied to reverse transcribe 2 μg of total RNA. qRT-PCR was conducted using a Bio–Rad CFX96 system with SYBR green to determine the mRNA level of a gene of interest; the gene expression level was normalized to the GAPDH mRNA level using the 2 − ΔΔCt method. RNase R treatment (2 U/mg) was performed for 15 min at 37 °C. The primers we used for PCR are listed in Supplementary Table 1.

### Luciferase reporter assay

The 3’ untranslated region (UTR) of PD-L1 with wild-type (WT) or mutant (Mut) miRNA-responsive elements was cloned into the psiCHECK-2 vector (Promega, Madison, WI, USA). Cells were plated in 24-well plates and transfected with the constructed cDNA using Lipofectamine 3000 transfection reagent (Invitrogen, Carlsbad, CA) according to the manufacturer’s instructions. Luciferase activity was measured 36–48 h after transfection with a Dual-Luciferase Assay (Promega) according to the manufacturer’s manual.

### The circRNA/mRNA-miRNA pull-down assay

Collected cell lysates with 1 µl of RNase inhibitor added were mixed with 500 pM biotinylated antisense oligos against the PD-L1 mRNA (5’-ATG TCA GTG CTA CAC CAA GGC-3’) or circ_0001005 sequence (5’-TAA GAT GTC GTT GTA TAA CTA AGT AGA CTT TTG ATG T-3’) after specified treatments were administered and then rotated overnight at 4 °C. Streptavidin Agarose beads (10 µl) were added, and the lysate mixture was rotated for 1 h at 4 °C and then washed with RIP buffer five times. Total RNA was extracted following the manufacturer’s protocol. After reverse transcription, qPCR analysis was applied to detect the miRNAs pulled down by PD-L1 and circ_0001005.

### In vivo studies

For in vivo studies, male, athymic BALB/c nude mice (6 weeks old) were randomly distributed into four groups (*n* = 6 per group). TCC-SUP cells with or without NK-92 cells with Effector cells: Target cells ratio = 1:5 (E.T. ratio = 1.5) were injected subcutaneously to establish the xenograft model. Two weeks after implantation, when the tumors were observed, mice were intraperitoneally treated with 30 mg/kg Enz or DMSO every other day for 4 weeks until mice met treatment endpoints. The average gross tumor sizes of each group were monitored during the treatment. Tumors were measured by calipers every week to calculate volume changes. A week after treatments, mice were sacrificed and tissues removed for measurements, IHC, and qPCR studies. The animal experiments were approved and conducted under the supervision of the University Committee on Animal Resources of University of Rochester Medical Center.

### Statistical analysis

Experiments were repeated independently at least three times with data points completed in triplicate. Results are shown as the mean ± S.D. Statistical significance was determined using Student’s *t*-test or two-way ANOVA with SPSS 22 (IBM Corp., Armonk, NY) or GraphPad Prism 6 (GraphPad Software, Inc., La Jolla, CA). P values less than 0.05 were considered statistically significant (*P < 0.05, **P < 0.01, and ***P < 0.001).

## Results

### AR could affect PD-L1 expression in BCa cells

**Fig. 1:**
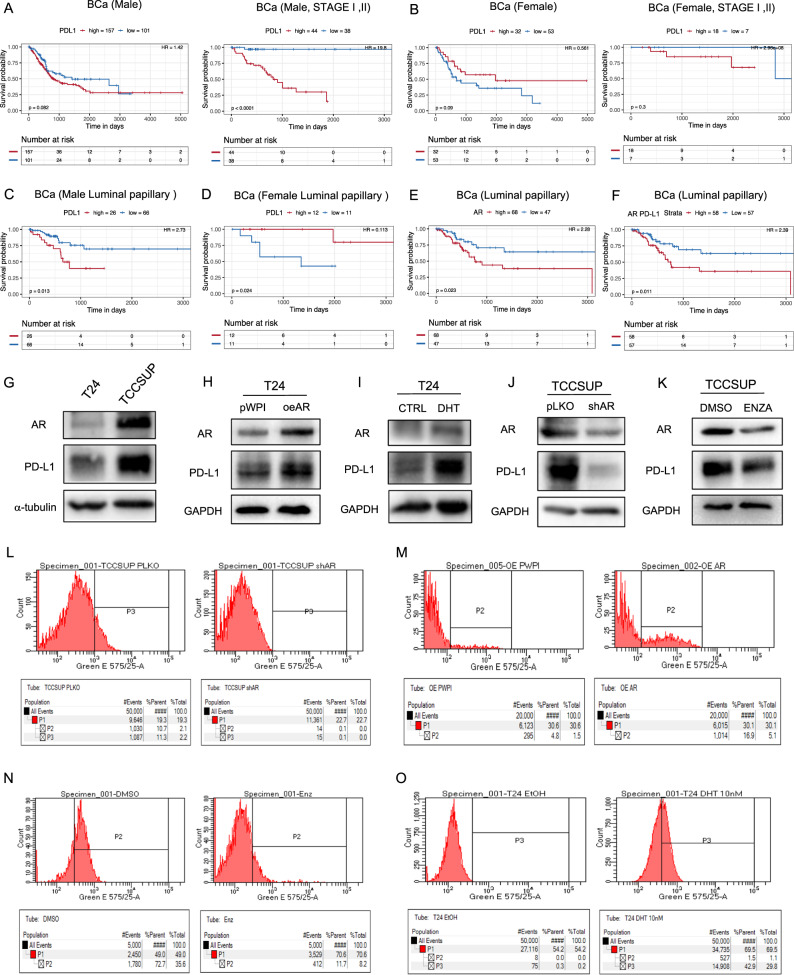
Regulation of PD-L1 expression by altering androgen receptor (AR) in BCa cells. **A** The overall survival hazard ratio of high/low PD-L1 level in male BCa patients from all stages (left) and earlier stages (stage I and II) (right). **B** The overall survival hazard ratio of high/low PD-L1 level in female BCa patients from all stages (left) and earlier stages (stage I and II) (right). **C** The overall survival hazard ratio of high/low PD-L1 level in male patients with luminal papillary BCa. **D** The overall survival hazard ratio of high/low PD-L1 level in female patients with luminal papillary BCa. **E** The survival hazard ratio of high/low AR level in patients with luminal papillary subtype. **F** The survival hazard ratio of both PD-L1 and AR high/low level in patients with luminal papillary subtype. **G** The AR and PD-L1 expressions in T24 and TCC-SUP cell lines. **H**, **I** The AR and PD-L1 expression was measured after overexpressing AR (oeAR) in T24 cells (**H**) and knocking down AR (shAR) in TCC-SUP cells (**I**). **J**, **K** The PD-L1 expression was measured after T24 cells were treated with 10 nM DHT (**J**) or in TCC-SUP cells were treated with 10 μM anti-androgens Enz (**K**). **L**, **M** The membrane PD-L1 expression level was tested by flow cytometry assay after changing (**L**, shAR; **M**, oeAR) AR expression. **N**, **O** Membrane PD-L1 expression was measured after treating TCC-SUP cells with or without Enz (**N**) or treating T24 cells with or without DHT (**O**). Survival analysis performed in TRGAted utilizes the survival (v2.41-3) and survminer (v0.4.2) R packages. Optimal cutpoints are based on the surv_cutpoint function in the survminer package, finding the lowest log-rank p-value with the minimum proportional comparison set to 15% versus 85% of samples.

Previous studies showed AR acting as an oncogene could increase BCa invasion and cisplatin-resistance [7]. However, whether AR in BCa cells can influence antitumor immunity by regulating immune cell killing efficiency has not been studied. We focused on the classic critical immune checkpoint molecule PD-L1, a key molecule immunotherapy [7]. According to data from the TCGA, high expression of AR signaling-involved genes was detected in the luminal papillary mRNA subtype of BCa [28]. The latest study on the gender difference in BCa highlighted that male patients were more likely to develop tumors of luminal papillary subtype than female patients [29]. By utilizing the TRGAted to analyze the online TCGA database [30], we found that the prognostic value of PD-L1 in BCa was largely different between male patients and female patients, showing a tendency toward lower survival in male patients with higher PD-L1 expression levels, it showed more significant in male patients from earlier clinical stages (stage I and II). While there was an opposite tendency in female patients (Fig. 1A, B). Male patients with no distant metastasis showed a much stronger negative correlation between PD-L1 expression and survival, while female patients presented the opposite tendency (Fig. S1A). In the luminal papillary subtype, a higher level of PD-L1 was significantly associated with worse survival in male patients (Fig. 1C). In female patients, the prognostic value of PD-L1 was completely opposite that in male patients (Fig. 1D). Then, we analyzed the survival hazard ratio of the AR protein level in BCa, which showed that higher AR expression in patients correlated with worse progression-free survival in the luminal papillary subtype (Fig. 1E) and the luminal infiltrated subtype (Fig. S1B). In the luminal papillary subtype, patients with high PD-L1 and AR levels had worse progression-free survival (Fig. 1F). Additionally, in patients with luminal subtype BCa, high levels of both AR and PD-L1 indicated a lower overall survival probability (Fig. S1C).

To test whether antiandrogens/androgen impacts PD-L1 expression, we performed Western blot assays with the human BCa cell lines T24, which has lower AR expression, and TCC-SUP, which has higher AR expression, to detect PD-L1 expression (Fig. 1G). Altering AR expression in T24 cells by transducing AR-cDNA to overexpress AR (oeAR) and adding the androgen DHT substantially upregulated PD-L1 expression (Fig. 1H, I), while knocking down AR expression and activity in TCC-SUP cells by transducing AR-shRNA (shAR) and treating the cells with the antiandrogen Enz downregulated PD-L1 expression (Fig. 1K). Moreover, treatment with another antiandrogen, such as HF or the AR-degradation enhancer ASC-J9®, also decreased PD-L1 expression (Fig. S1D). Since membrane PD-L1 plays a functional role in facilitating immune escape by binding to its ligand PD-1 on immune cells, flow cytometry assays were applied to test membrane PD-L1 expression after altering AR expression. As expected, membrane PD-L1 expression was upregulated and downregulated by increasing and decreasing AR expression, respectively (Fig. 1L, M). Treatment with the antiandrogen enzalutamide (Enz) decreased membrane PD-L1 expression in TCC-SUP cells (Fig. 1N). In contrast, treatment with the androgen DHT notably increased membrane PD-L1 expression in T24 cells (Fig. 1O). Together, the data from Fig. 1g-o and S1d indicate that AR can regulate PD-L1 expression in BCa cells.

### The upregulation of PD-L1 by AR in BCa cells could affect the tumor-killing efficacy of NK cells

**Fig. 2:**
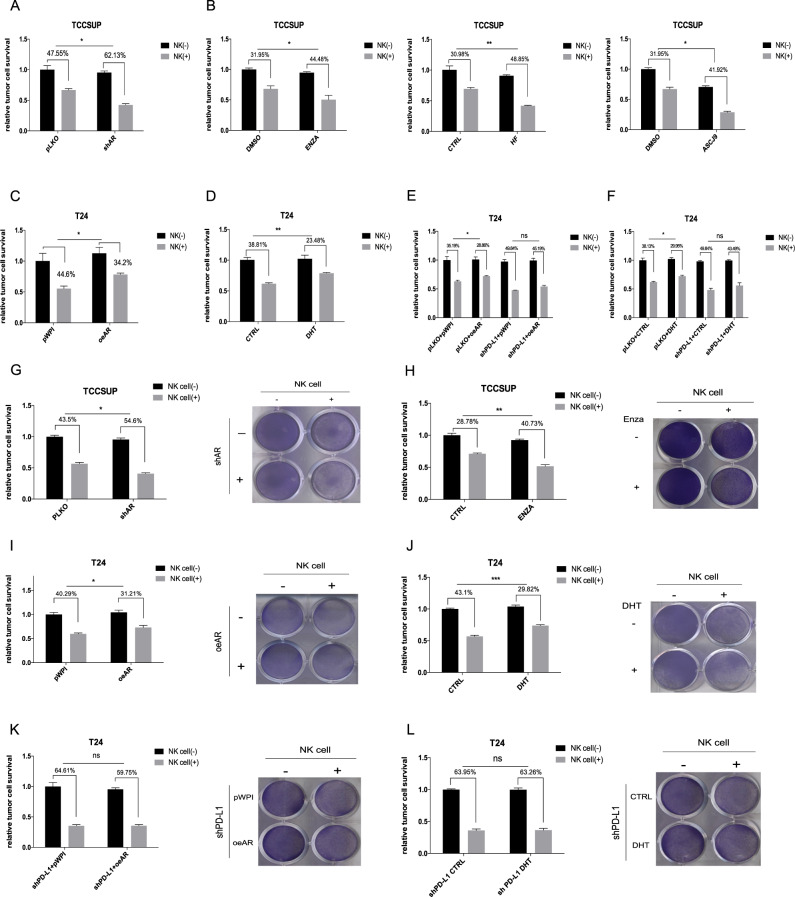
NK cell killing efficacy against BCa cells impacted by AR-regulated PD-L1 expression in BCa cells. **A** NK cell-mediated tumor cell killing assay. TCC-SUP pLKO and shAR cells co-cultured with or without NK cells (E:T ratio is 5:1) for 48 h were subjected to MTT assay. **B** NK cell-mediated tumor cell killing assay was tested in TCC-SUP cells treated with DMSO and anti-androgens Enz (10 μM) or hydroxyflutamide (10 μM), as well as ASC-J9^®^ (5 μM). **C** NK cell-mediated tumor cell killing assay was tested in T24 pWPI and oeAR cells. **D** NK cell-mediated tumor cell killing assay was tested in T24 cells treated with ethanol as control (CTRL) and DHT (10 nM). **E** NK killing assay was performed with T24 pLKO cells and T24 shPD-L1 cells with and without over-expressed AR. **F** NK killing assay was tested in T24 pLKO cells and T24 shPD-L1 cells with and without DHT treatment (10 nM). **G** TCC-SUP pLKO and shAR cells co-cultured with or without NK cells (E:T ratio is 5:1) for 48 h were subjected to crystal violet staining. **H** NK cell-mediated tumor cell killing assay was tested in TCC-SUP cells treated with DMSO and anti-androgens Enz (10 μM) by crystal violet staining. **I** NK cell-mediated tumor cell killing assay was tested in T24 pWPI and oeAR cells by crystal violet staining. **J** NK cell-mediated tumor cell killing assay was tested in T24 treated with and without DHT (10 nM) by crystal violet staining. **K** NK killing assay was tested in T24 shPD-L1 with and without over-expressed AR by crystal violet staining. **L** NK killing assay was tested in T24 shPD-L1 with and without DHT treatment (10 nM) by crystal violet staining. For **G**–**L** quantitations are on the left. The values are the means ± SD from at least 3 independent experiments. *P < 0.05, **P < 0.01, ***P < 0.001, ns = not significant.

To further determine whether the regulation of PD-L1 via AR affects immune cell killing efficacy against BCa cells, BCa cells were incubated with or without NK cells. The efficacy of NK cell killing of BCa cells was measured with MTT to test whether the tumor cell survival probability was reduced and/or enhanced following changes in AR expression and/or activity. For TCC-SUP cells with AR knockdown (shAR) **(**Fig. 2A) treated with an antiandrogen (Enz, HF, or ASC-J9) (Fig. 2B), the NK cell killing efficacy against these BCa cells was enhanced, while for T24 cells, AR overexpression (oeAR) (Fig. 2C) and DHT treatment (Fig. 2D) could reduce the killing efficacy. To prove that AR impacts this killing efficacy by altering PD-L1 expression, we knocked down PD-L1 expression via PD-L1-shRNA (shPD-L1) introduction and showed a reversal of the oeAR-dampened NK cell killing efficacy (Fig. 2E). Treatment with DHT also showed a similar reversal (Fig. 2F). Western blot assays were used to validate the successful knockdown of PD-L1 expression by shPD-L1 in TCC-SUP cells (Fig. S1E).

Moreover, we performed crystal violet staining of surviving BCa cells to assess NK cell killing efficacy. For TCC-SUP cells, knocking down AR or administering Enz treatment each improved the NK cell killing efficacy (Fig. 2G, H). Over-expressing AR and administering DHT treatment both impeded the efficacy of NK cell killing of T24 cells (Fig. 2I, J), which could be reversed by shPD-L1 (Fig. 2K, L).

The data shown in Fig. 2A–L indicate that AR can affect the efficacy of NK cell killing of tumor cells by altering PD-L1 expression.

### AR regulates PD-L1 expression post-transcriptionally by altering circ_0001005 expression to impact NK cell killing efficacy

**Fig. 3:**
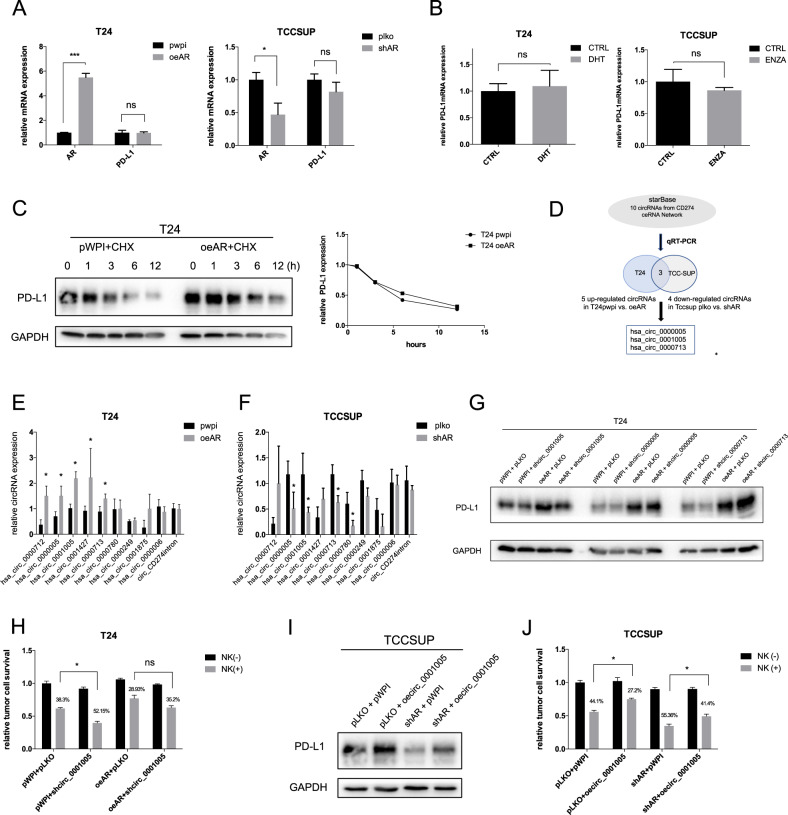
AR promotes PD-L1 expression *via* increasing circ_0001005, a post-transcriptional mechanism. **A** The qRT-PCR assay for PD-L1 mRNA level in T24 cells (left) transfected with oeAR versus pWPI and TCC-SUP cells (right) transfected with shAR versus pLKO. **B** The mRNA levels of PD-L1 in T24 cells (left) treated with/without DHT and TCC-SUP cells (right) treated with/without Enz (ENZA). **C** Western blot was used to determine PD-L1 protein stability in T24 cells transfected with oeAR versus pWPI plus CHX, quantitation on the right. **D** The illustration of screening circRNA candidates. Based on the online database, 9 circRNAs from ceRNA network and 1 circRNA created by PD-L1 intron may be involved in the regulation of PD-L1 expression. Among these 10 circRNAs 5 were upregulated in oeAR and 4 were downregulated in shAR by qRT-PCR, 3 of the circRNAs could be the functional candidates shown by merging. **E**, **F** The qRT-PCR was performed to evaluate the expression of the 10 circRNAs in T24 cells transfected with oeAR or pWPI (left) and in TCC-SUP cells transfected with shAR or pLKO (right). **G** PD-L1 expression was determined by Western blot after transducing pLKO-shcirc_0000005, pLKO-shcirc_0001005, or pLKO-shcirc_0000713 into T24 cells with pWPI or oeAR. **H** NK cell killing assays were conducted to test the function of shcirc_0001005 in T24-pWPI and T24-oeAR cells by MTT. **I** PD-L1 expression was determined by Western blot after transducing pWPI vs oecirc_0001005 in TCC-SUP-pLKO and TCC-SUP-shAR cells. **J** NK cell killing assays were applied to test the function of oecirc_0001005 in TCC-SUP-pLKO and TCC-SUP-shAR cells. The values are the means ± SD from at least 3 independent experiments. *P < 0.05, ns=not significant.

The mRNA expression of PD-L1 was tested after overexpression or knockdown of AR in the T24 and TCC-SUP cell lines, respectively, and showed no significant change (Fig. 3A). Treatment with an androgen (DHT) or antiandrogen (Enz) did not change PD-L1 ex-pression at the mRNA level (Fig. 3B). To further dissect how AR regulates PD-L1 expression at the protein level, T24 cells transfected with pWPI or oeAR were treated with cycloheximide (CHX) for designated periods of time to detect the decay of the PD-L1 protein. However, the results showed no significant differences (Fig. 3C), which indicated that protein stability was not affected. Thereafter, we hypothesized that AR signaling may regulate PD-L1 protein levels indirectly via a noncoding RNA pathway(s). Based on a previous study in our laboratory, which showed that AR may regulate circRNA expression to further affect functions via the RNA-editing gene ADAR2 in BCa [31], we tested the involvement of circRNAs as competing endogenous RNAs (ceRNAs) in the regulation of PD-L1 expression. To evaluate this possibility, we examined 10 circRNAs, including 9 PD-L1-related ceRNAs found online using the database starBase: hsa_circ_0000712, hsa_circ_0000005, hsa_circ_0001005, hsa_circ_0001427, hsa_circ_0000713, hsa_circ_0000780, hsa_circ_0000249, hsa_circ_0001875, and hsa_circ_0000006; we also tested circ_PD-L1 intron, which is produced from the PD-L1 intron predicted by the online database CSCD [32]. We examined the impact of AR on the expression of these 10 circRNAs by qRT–PCR. The results showed that the expression of three of the circRNAs (hsa_circ_0000005, hsa_circ_0001005, and hsa_circ_0000713) was consistently changed by AR overexpression or knockdown (Fig. 3D–F).

To further test whether AR regulates any of these 3 circRNAs to increase PD-L1 expression and modulate NK cell killing efficacy in BCa, shRNAs targeting the specific 5’-3’ splice junctions of these 3 circRNAs (sh-circRNA) were constructed (Fig. S2A). We carried out rescue experiments using these 3 sh-circRNAs and found that knockdown of circ_0001005 could reverse the oeAR-increased PD-L1 protein expression in T24 cells (Fig. 3G). Moreover, knocking down circ_0001005 facilitated NK cell killing of BCa cells, which could be reversed by the function of oeAR (Fig. 3H).

To verify the circular form of circ_0001005, we applied qRT–PCR to test the levels of the circRNA and GAPDH mRNA in TCC-SUP and T24 cells treated with RNase R and showed that RNase R treatment did not decrease the level of circ_0001005 but did reduce the GAPDH mRNA level (Fig. S2B). Since RNase R effectively digests linear RNAs leaving only circular RNA behind, it greatly facilitates the identification of circular RNAs. Next, according to the structure of circ_0001005, we constructed pWPI-oecirc_0001005 for further experiments (Fig. S2C-D). Consistently, overexpressing circ_0001005 in TCC-SUP cells dampened NK cell killing efficacy and partially reversed shAR prompted NK cell killing efficacy by up-regulating the expression of PD-L1 (Fig. 3I, J).

### AR reduces circ_0001005 expression by regulating ADAR2

**Fig. 4:**
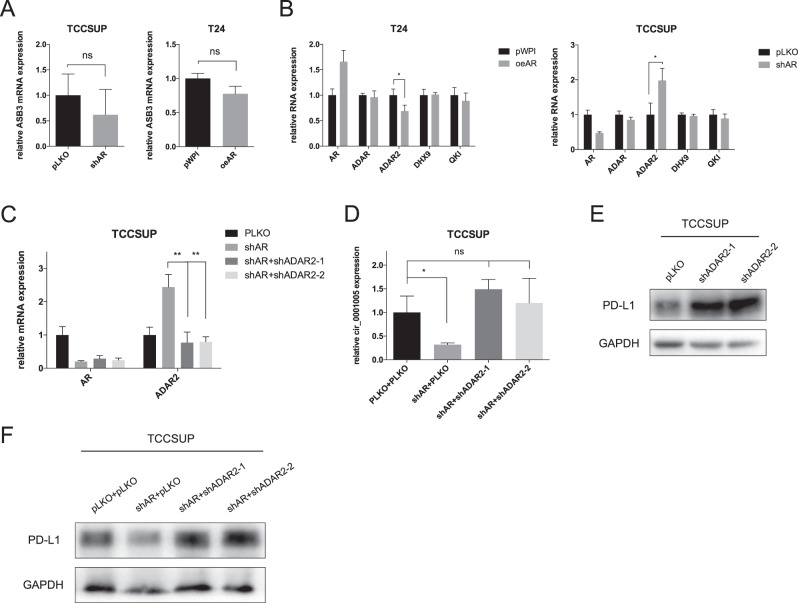
AR up-regulates circ_0001005 through the RNA editing gene ADAR2. **A** The mRNA level of ASB3 was measured in TCC-SUP shAR vs pLKO.1 (left) and T24 oeAR vs pWPI vector (right) cells using qRT-PCR. **B** The 4 RNA editing genes (ADAR, ADAR2, DHX9, and QKI) were tested by qRT-PCR after over-expressing AR (oeAR) in T24 cells (left) and knocking down AR (shAR) in TCC-SUP cells (right). **C** The efficacy of shAR and the two shRNAs of ADAR2 (shADAR2-1 and shASAR-2) was detected by qRT-PCR. **D** The expression of circ_0001005 was measured after transducing pLKO, shAR, shAR-shADAR2-1 and shAR-shADAR-2 into TCC-SUP cells. **E** The expression of PD-L1 was detected after knocking down shADAR2 in TCC-SUP cells. **F** The PD-L1 expression was tested by western blot in TCC-SUP cells transfected with pLKO, shAR, shAR-shADAR2-1, or shAR-shADAR-2. The values are the means ± SD from at least 3 independent experiments. *P < 0.05, **P < 0.01, ***P < 0.001, ns = not significant.

AR could transcriptionally regulate ADAR2 expression in BCa cells, which was validated by chromatin immunoprecipitation (ChIP) and luciferase reporter assays per-formed with the UMUC3 and J82 cell lines in previous research by our laboratory [33]. First, to exclude the possibility that AR could alter the target circRNA by regulating its corresponding host gene, we performed a qRT–PCR assay to test the expression of the circRNA parental gene ASB3 after increasing or decreasing AR expression. The results showed that AR did not significantly change the ASB3 mRNA level (Fig. 4A) in either TCC-SUP or T24 cells. Therefore, we reverified the expression of the 4 reported RNA-editing genes, ADAR, ADAR2, DHX9, and QKI, that were previously evaluated after overexpressing AR in T24 cells and knocking down AR in TCC-SUP cells. Our results showed that ADAR2 could be regulated by AR, as expected (Fig. 4B), in both cell lines. In addition, using constructed shRNAs specific for ADAR2, shADAR2#1 and shADAR2#2, could offset the enhancing effect of shAR on ADAR2 expression (Fig. 4C). We performed interference experiments using these two shADAR2 sequences to effectively reverse the decrease in circ_0001005 expression induced by shAR (Fig. 4D). Next, we confirmed that shADAR2#1 and shADAR2#2 could increase the PD-L1 level, which supports our hypothesis that ADAR2 can control cirASB3_016 to regulate PD-L1 (Fig. 4E). Moreover, our data revealed that shAR could downregulate PD-L1 expression, and this effect could be partially reversed in TCC-SUP cells by shADAR2 (Fig. 4F).

### AR/ARAR2-regulated circ_0001005 function via miRNA signaling to increase PD-L1 expression

**Fig. 5:**
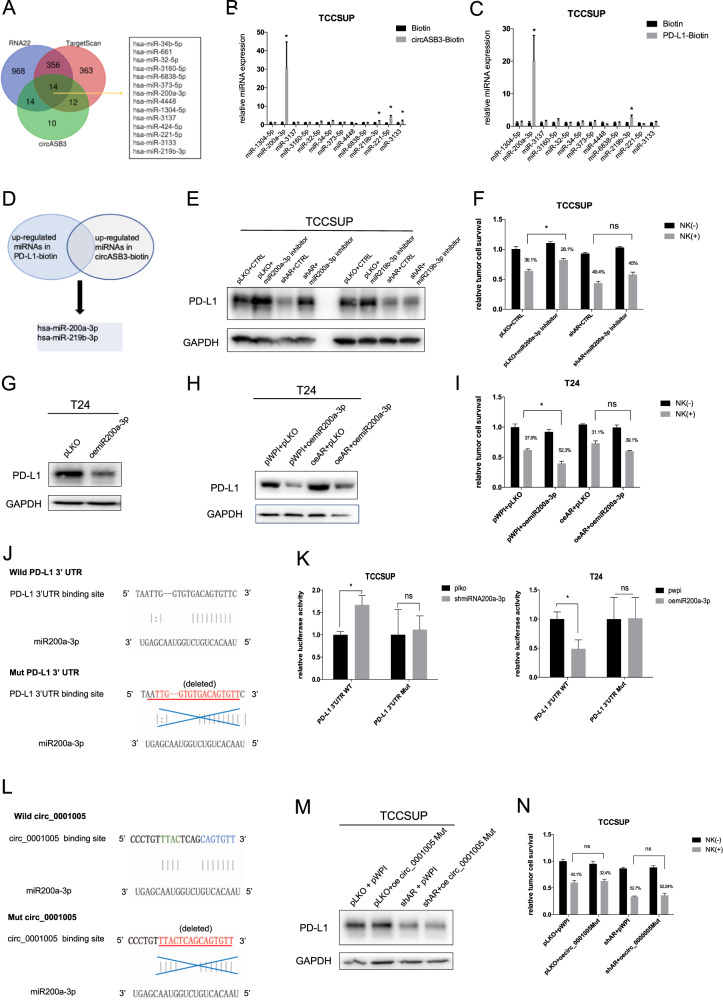
Mechanism dissection: Circ_0001005 up-regulates PD-L1 expression *via* competitive binding with miR-200a-3p. **A** Bioinformatics analysis of 14 miRNAs that are potentially associated with PD-L1 and regulated by circ_0001005. **B** Using circ_0001005-biotin to pull-down these 14 miRNAs to show the expression of miRNAs by qRT-PCR. **C** Using PD-L1-biotin to pull-down these 14 miRNAs to show the expression of miRNAs. **D** 2 miRNAs were functional, as shown by merging the results of the PD-L1-biotin and circASB3-biotin assays. **E** The western blot performed to determine PD-L1 expression after adding pLKO-miR200a-3p inhibitor/pLKO-miR219b-3p inhibitor into TCC-SUP cells transduced with pLKO or shAR. **F** NK killing assay using MTT was tested in TCC-SUP cells transfected with pLKO-miR200a-3p inhibitor and pLKO or shAR. **G** The expression of PD-L1 was detected after over-expressing miR200a-3p in T24 cells. **H** The expression of PD-L1 was measured in T24 cells transfected with pLKO-oemiR200a-3p and pWPI or oeAR. **I** NK killing assay using MTT was tested in T24 cells transfected with pLKO-oemiR200a-3p and pWPI or oeAR. **J** Wild type (Wild) and mutant (Mut) target sites of miR-200a-3p to PD-L1 3’UTR were designed. **K** Luciferase reporter activity was detected after transducing wild type (WT) and Mut PD-L1 3’UTR reporter in TCC-SUP cells comparing miR-200a-3p inhibitor vs vector (left) and in T24 cells comparing oemiR-200a-3p vs vector (right). **L** The structure of the binding sites between Wild circASB3 and miR-200a-3p (upper) and the construct of Mut circASB3 based on miR-200a-3p target site (lower). **M** The expression of PD-L1 was measured in TCC-SUP cells transfected with pWPI-oecirc_0001005 Mut and pLKO or shAR *. **N** NK killing assay using MTT was tested in TCC-SUP cells transfected with pWPI-oecirc_0001005 Mut and pLKO or shAR. “* The efficiency of oecirc_0001005 (WT and Mut) has been verified by qPCR.” The values are the means ± SD from at least 3 independent experiments. *P < 0.05, ns = not significant.

We considered that circ_0001005 could competitively sponge miRNAs to further modulate PD-L1 expression. The results of Ago2 pull-down assays supported our hypothesis that miRNAs were involved (Fig. S3A). We identified 14 microRNA candidates (miR-34b-5p, miR-661, miR-32-5p, miR-3160-5p, miR-6838-5p, miR-373-5p, miR-200a-3p, miR-4448, miR-1304-5p, miR-3137, miR-424-5p, miR-221-5p miR-3133, and miR-219b-3p) by using the online bioinformatic prediction databases RNA22 (https://cm.jefferson.edu/rna22/) [34], TargetScan (http://www.targetscan.org/vert_72/) [35], and circBank [36, 37] to predict circ_0001005-related miRNAs with the potential for PD-L1 3’UTR binding (Fig. 5A). We applied RNA pull-down assays to screen the potential miRNA candidates that could bind to both the PD-L1 3’UTR and circ_0001005 using biotinylated oligonucleotides against the 3’UTR of PD-L1 and circ_0001005 in TCC-SUP cells. Biotinylated circ_0001005 pulled down 4 of the 12 miRNAs, and biotinylated PD-L1 pulled down 2 miRNAs, which indicated that 2 of the miRNAs, miR-200a-3p and miR219b-3p, could be detected in both the biotinylated PD-L1-3’UTR and biotinylated circ_0001005 pull-down complexes (Fig. 5B–D). Then, we constructed miRNA inhibitors for the 2 miRNAs and separately transduced them into TCC-SUP cells. The miR200a-3p inhibitor but not the miR219b-3p inhibitor significantly increased PD-L1 expression and partially reversed the shAR-induced de-crease in the PD-L1 level (Fig. 5E and Fig. S3B). Moreover, treatment with the miR200a-3p inhibitor could reduce NK cell killing of tumor cells, which could reverse the shAR-induced increase in NK cell killing efficacy (Fig. 5F). Next, we transduced oemiR200a-3p into T24 cells to confirm that miR-200a-3p could re-verse the AR-induced increase in PD-L1 expression and oeAR-induced reduction in NK cell killing efficacy (Fig. 5G–I and SFig. 3 C).

To elucidate the mechanism by which miR-200a-3p modulates PD-L1 expression, we identified the potential structures of miR-200a-3p target sites in the 3’UTR of PD-L1 using the RNA22 website (https://cm.jefferson.edu/ rna22/). Then, we cloned the WT 3’UTR of PD-L1 and a Mut 3’UTR of PD-L1 with the miRNA target site deleted into the psiCHECK-2 vector (Fig. 5J).

After transfection of the psiCHECK-2 luciferase reporters carrying the WT or Mut PD-L1 3’UTR into TCC-SUP cells to compare shmiR-200a-3p vs. vector and into T24 cells to compare oemiR-200a-3p vs. vector, we performed luciferase reporter assays to examine the interaction between miR-200a-3p and the PD-L1 mRNA transcript. The results revealed that either a decrease or an increase in miR-200a-3p could significantly alter the luciferase reporter activity of the WT PD-L1 3’UTR, as expected, but had little effect on the Mut PD-L1 3’UTR in TCC-SUP and T24 cells (Fig. 5K).

To further validate that miR-200a-3p was sponged by circ_0001005 to produce the observed outcome, we constructed circ_0001005 Mut, which had a mutation in the miR200a-3p binding site (Fig. 5L, Fig. S3D). Overexpression of circ_0001005 WT increased PD-L1 expression, while overexpression of circ_0001005 Mut did not reverse either the shAR-mediated upregulation of PD-L1 ex-pression in BCa cells or the shAR-induced reduction in the NK cell killing efficacy against BCa cells (Fig. 5M, N).

Together, the results shown in Fig. 5A–N and Fig. S3A–D suggest that circ_0001005 can sponge miR-200a-3p to increase PD-L1 expression, consequently impacting NK cell killing efficacy.

### Preclinical study using an in vivo mouse model to confirm the role of the AR/circ_0001005/PD-L1 axis in impacting NK cell antitumor efficacy

**Fig. 6:**
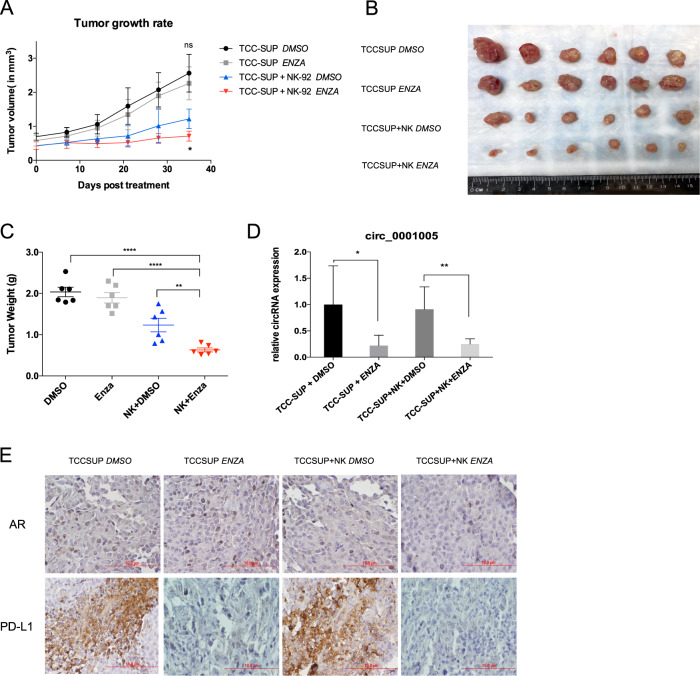
Preclinical study using in vivo mouse BCa model showing the AR regulated circ_0001005 plays critical roles in regulating NK cells anti-tumor efficacy. **A** TCC-SUP cells with or without NK cells were injected subcutaneously into nude mouse to establish the xenograft model, and Enz (30 mg/kg) or DMSO was intraperitoneally administered. The tumor growth rates were measured and compared among the four groups. **B** Images show the tumors acquired after mice were sacrificed (*N* = 6). **C** Tumor weights of each group were measured and compared. **D** The qRT–PCR was performed to test the circ_0001005 level in the four groups using mouse tumor tissues. **E** Representative immunohistochemistry (IHC) images detecting PD-L1 and AR in xenografted tumors of each group. (Scale bars: 10 um) The values are the means ± SD from at least 3 independent experiments. *P < 0.05, **P < 0.01, ****P < 0.001.

To further confirm that targeting AR through AR/circ_0001005/PD-L1 signaling could impact NK cell antitumor efficacy, we conducted a preclinical experiment using a xenograft BALB/c nude mouse model. TCC-SUP cells with or without NK-92 cells were injected subcutaneously into mice, which were then treated with Enz or DMSO as a control. The mice were divided into four groups: the TCC-SUP DMSO-treated group, TCC-SUP Enz-treated group, TCC-SUP + NK cell DMSO-treated group, and TCC-SUP + NK cell Enz-treated group. Lower xenograft tumor growth rates and tumor weights were found in the Enz-treated TCC-SUP + NK cell group than in the TCC-SUP + NK cell DMSO-treated group. In xenografted mice injected with TCC-SUP cells, the tumor growth rates and tumor weights were not significantly decreased by treatment with Enz (Fig. 6A–C). Tumor tissues from sacrificed mice were evaluated by qRT–PCR, and the results con-firmed that the expression of circ_0001005 was decreased by Enz treatment (Fig. 6D). Furthermore, immunohistochemical staining of tumor tissues revealed that the expression of PD-L1 was decreased by targeting AR with Enz (Fig. 6E).

**Fig. 7:**
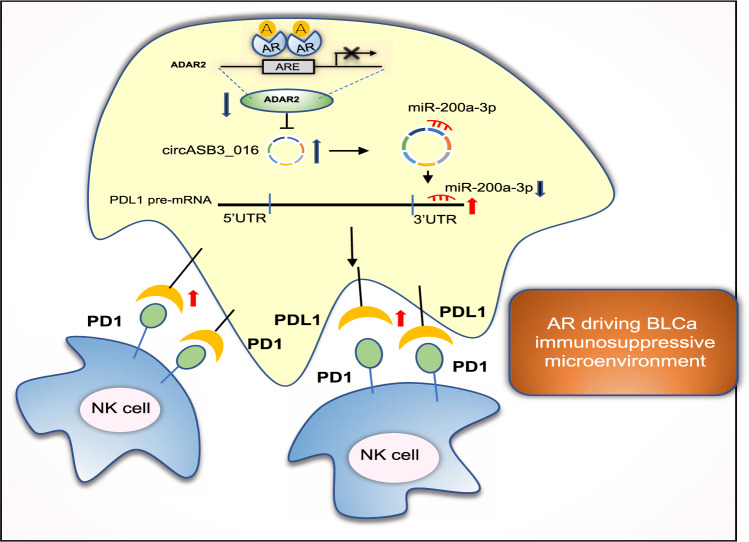
Illustration of AR/ADAR2/circ_0001005/miR-200a-3p/PD-L1 signaling in driving BCa immunosuppressive microenvironment. Upon ligand binding with androgen (A), the androgen receptor (AR) interacts with an androgen response element (ARE) in the promoter region of the RNA editing gene ADAR2, decreasing its expression. This in turn increases circ_0001005 levels, thus elevating the sponging of miR-200a-3p, to increase PD-L1 expression. Elevated levels of PD-L1 impede NK cells killing towards BCa cells, assisting tumor cells’ immune escape and driving immunosuppressive microenvironment.

Together, the results presented in Fig. 6A–E demonstrate that targeting AR/circ_0001005/PD-L1 signaling improves NK cell antitumor efficacy in vivo (Fig. 7).

## Discussion

Since the occurrence of BCa in patients shows apparent sex differences, sex hormones, including androgen and estrogen, have been suggested to perform crucial roles [2, 38]. AR has been shown to play important roles in the initiation and progression of BCa [26]. Additionally, its impact on the tumor immune environment has been suggested by previous papers [11, 12]. Recent studies have proposed that the activation of the AR pathway is increased in the luminal papillary (PAP), squamous (SCC) and mesenchymal-like (MES) subtypes of urothelial BCa. Although the MES and SCC subtypes show upregulated immunosuppressive PD-1 and CTLA-4 signaling, according to IMvigor210 data, they do not significantly benefit from anti-PD-L1 therapy [39]. Additionally, activated TGF-β signaling, as well as increased M2 macrophage infiltration, also contributes to the negative influence of these immune checkpoints on the tumor immune microenvironment to further impede the response to immunotherapy [39, 40]. Previous studies on AR in BCa have identified roles in tumor proliferation, progression, and metastasis, and AR inhibitors and the degradation enhancer ASC-J9^®^ could be viable options for BCa therapy [41, 42]. In our study, we investigated whether the increased AR signaling induced by over-expression and treatment with an exogenous androgen could dampen the NK cell killing efficiency against tumor cells by upregulating the classic immunosuppressive molecule PD-L1. Herein, NK cells, which play a part in innate immunity, were used in killing assays. Although T cells have recently attracted attention in antitumor immunity because of their connection to immune checkpoint therapy, NK cells have also gained increasing attention related to therapeutic strategies harnessing NK cells for metastasis control, in which immune checkpoint inhibitors targeting PD-1, CTLA4, or PD-L1 may boost NK cell effector functions [43, 44].

PD-L1 is known to play an immunosuppressive role in tumors and to contribute to immune escape to facilitate the initiation and progression of cancer. In addition to cytokines that can induce and maintain PD-L1 expression [45, 46], the intrinsic oncogenic signaling pathway can regulate PD-L1 expression [47]. Many previous studies on AR in BCa have demonstrated the oncogenic role of AR that impacts both initiation and progression. Moreover, the role of AR in modulating adaptive immunity has also been explored [48, 49]. In our study, we explored whether AR can promote BCa development by upregulating PD-L1 expression and whether blocking AR signaling facilitates NK cell killing of BCa cells. Increased NK cell cytotoxicity has been related to a better prognosis and lower metastatic risks in several cohorts of cancer patients [50–52]. Despite the knowledge on classic NK cell activating receptors, the immunosuppressive receptors PD-1 and CTLA-4 have gained increasing attention as targets for improving NK cell-mediated immunity and combination therapies, which have been approved for evaluation in clinical trials [43, 44]. Herein, we utilized AR inhibitors to diminish PD-L1 expression on tumor cells via the novel ADAR2/circ_0001005/miR200a-3p signaling pathway, thereby enhancing NK cell-based immunotherapy. In the intricate human setting, AR-induced PD-L1 expression on tumor cells may impede the function of immune cells targeted by PD-L1, thus weakening antitumor immunity. Therefore, we suspected that AR might facilitate tumor cell escape from immune surveillance via the PD-L1-induced suppressive state in cytotoxic immune cells. Combining the present study with previous research on the protumor role of AR in BCa helped us establish a broader understanding of how AR performs its roles to initiate and promote tumorigenesis.

Studies on circRNAs in cancer have unveiled their noteworthy roles in contributing to and influencing tumor development, progression, and metastasis. circRNAs have a covalently closed loop structure, making them resistant to ribonucleases. Due to their structural characteristics and properties, circRNAs participate in multiple biological processes through different functional mechanisms, such as miRNA sponging, transcriptional regulation or splicing, RNA-binding protein interactions and modulation of protein translation [53, 54]. In BCa, research on the regulatory roles of circRNAs has focused on effects on tumor proliferation, invasion, and migration [55, 56]. A published paper reported that transfection of a purified synthetic circRNA in vitro could contribute to substantial induction of innate immune genes [57]. Another study investigated whether circRNAs expressed in macrophages could affect the expression of genes encoding inflammatory molecules [58], implying that circRNAs may play critical roles in immune regulation; however, these topics remain to be further explored. In a previous study, AR was shown to regulate circRNAs via the RNA-editing gene ADAR2 to further exert its biological function [31]. In our present study, we examined whether AR-regulated ADAR2 could increase PD-L1 expression by regulating circ_0001005 to help BCa cells escape immune cell killing. Through mechanistic studies, we found that circ_0001005 regulates PD-L1 expression through competitively sponging miR200a-3p, one of the classic regulatory mechanisms. Our investigation of an innovative regulatory mechanism for PD-L1 expression mediated through circ_0001005 represents a pioneering way to identify prospective strategies for BCa therapy.

As the most classic and extensively studied noncoding RNAs, miRNAs are clearly involved in regulating gene expression, existing as free molecules or packaged in extracellular vesicles found in human blood and bodily fluids [59–62]. Many studies have verified the crucial roles of miRNAs in NK cell differentiation/development [63, 64]. In various human cancers, miRNAs can play oncogenic or tumor-suppressive roles by modulating NK cell antitumor activity to remodel the tumor microenvironment [65, 66]. Recent studies have demonstrated that some miRNAs can downregulate PD-L1 expression by directly targeting the PD-L1 3’UTR [67, 68]. Our results showed that miR-200a-3p binding to the 3’UTR of the PD-L1 mRNA transcript dramatically decreased PD-L1 expression. However, when miR-200a-3p was sponged by AR-regulated circ_0001005, the decrease in the level of this miRNA resulted in increased PD-L1 expression and thus impeded NK cell antitumor activity. Consistently, in malignant pleural mesothelioma, miR200 has been recognized as a tumor suppressor, contributing to the downregulation of PD-L1 expression [69]. Here, we found that the function of miR200a-3p could be affected by the sponging mechanism involving circ_0001005, which allowed this circRNA to impact PD-L1 ex-pression and the tumor immune microenvironment. Compared with previous work on miRNAs regulating PD-L1 expression, our study on circRNA-mediated regulation of PD-L1 is novel and promising. Given the burgeoning research on circRNAs, we may find that these noncoding RNAs notably influence tumor immune regulation.

## Conclusions

Overall, AR-regulated circ_0001005 could impact NK cell antitumor activity by altering PD-L1 expression in BCa, further illuminating that AR signaling promotes BCa initiation and progression. This study may refer to the influence of AR on the tumor immune microenvironment and provide a potential approach to target this newly identified pathway to better suppress BCa progression.

## Supplementary information

Supplementary figure legend

Supplementary methods

Supplementary figure

Supplementary table

## Data Availability

The datasets used and analyzed during the current study are available in the article and supplementary files, or available from the corresponding author on reasonable request.
