# Supplementary material for: Targeting the androgen receptor to enhance NK cell killing efficacy in bladder cancer by modulating ADAR2/circ_0001005/PD-L1 signaling
Source: Cancer Gene Ther. 2022 Aug 1;29(12):1988–2000. doi: 10.1038/s41417-022-00506-w (PMC9750871; doi:10.1038/s41417-022-00506-w)
Supplement: Supplementary file 1 — Supplementary figure legend [file 41417_2022_506_MOESM1_ESM.doc]

**Supplementary Figure 1.**

(A) The survival hazard ratio of high/low PD-L1 level in male (left) and female (right) non-metastasis BCa patients. (B) The survival hazard ratio of high/low AR level in male patients with luminal infiltrated subtype. (C) The survival hazard ratio of both PD-L1 and AR high/low level in male patients with luminal subtype. (D) The expression of PD-L1 was tested in TCC-SUP cells after treated with and without anti-androgen 10 μM hydro-flutamide (HF) (left) and with and without androgen receptor degrader ASC-J9® (5 μM) (right). (E) Western blot assay was used to confirm the knock down efficacy of shPD-L1 in TCC-SUP cells transfected with pLKO or shPD-L1. The values are the means ± SD from at least 3 independent experiments.

**Supplementary Figure 2.**

(A) The expression level of circRNA was measured by qRT-PCR after knocking down the three circRNA candidates. (B) The expression of hsa_circ_0001005 and GAPDH mRNA in T24 and TCC-SUP cells treated with or without RNase R was detected by qRT-PCR. (C) The schematic diagram of genomic location and splicing pattern of hsa_circ_0001005. (D) After transfecting BCa cells with and without pWPI-oehsa_circ_0001005, the level of hsa_circ_0001005 was tested by qRT-PCR. The values are the means ± SD from at least 3 independent experiments. *P<0.05, **P<0.01, ***P<0.001, ns=not significant.

**Supplementary Figure 3.**

(A) The Ago2 pull-down results indicated that oeAR-decreased PD-L1 mRNA level in the Ago2 complex, implying AR may modulate PD-L1 expression post-transcriptionally involving miRNAs. (B) The qRT-PCR was used to test the efficacy of miRNAs in TCC-SUP cells transfected with miR-300a-3p inhibitor or miR-219b-3p inhibitor. (C) The qRT-PCR was used to test the efficacy of miRNAs in T24 cells transfected with oemiR-300a-3p. (D) The expression level of circRNA was measured by qRT-PCR in TCC-SUP cells transfected with pWPI-oehsa_circ_0001005 WT or oehsa_circ_0001005 Mut. The values are the means ± SD from at least 3 independent experiments. *P<0.05.
