# Supplementary material for: Targeting the androgen receptor to enhance NK cell killing efficacy in bladder cancer by modulating ADAR2/circ_0001005/PD-L1 signaling
Source: Cancer Gene Ther. 2022 Aug 1;29(12):1988–2000. doi: 10.1038/s41417-022-00506-w (PMC9750871; doi:10.1038/s41417-022-00506-w)
Supplement: Supplementary file 2 — Supplementary methods [file 41417_2022_506_MOESM2_ESM.doc]

**MTT assay:** Adding 50 μL of 5 mg/mL MTT into each well and blank controls of media without cells. After incubating the plates for 2 h at 37 ◦C, media was removed and 150 μL DMSO added per well to dissolve the precipitate. The plates covered with foil were then placed on an orbital shaker for 15 min. The absorbance was measured at 570 nm.

**Crystal violet staining:** Place the plate with cells on ice and wash the cells twice with cold PBS. Fix the cells for 10 minutes with ice-cold 100% methanol. Aspirate methanol from the plates and and cover the cells with 0.5% crystal violet solution in 25% methanol. Incubate for 10 minutes at room temperature. Remove the crystal violet and wash the stained cells in water several times, until the dye stops coming off. The absorbance was measured at 570 nm.

**Immunohistochemistry (IHC) staining:** After tissue sample preparation, fixation, deparaffinization, hydration, antigen retrieval and blocking, the slices were incubated with primary antibodies in 3% BSA resolved in PBS at 4°C overnight, followed by biotinylated secondary antibodies (Vector Laboratories, Burlingame, CA, USA). The VECTASTAIN ABC peroxidase system and 3,3'-diaminobenzidine (DAB) kit (Vector Laboratories, Burlingame, CA, USA) were used for visualization. Brown staining characterized positive expression of the target protein. The percentage of positive cells was rated per high-power field (HPF) by 400× magnification as follows: 0 for sections with 1% positive cells; 1 for 1 to 25% positive cells; 2 for 26 to 50% positive cells; 3 for 51% to 75% positive cells; 4 for 76% to 100% positive cells. The staining intensity was graded as follows: weak intensity graded as 1, moderate intensity as 2, and high intensity as 3. Points for the percentage of positive cells and staining intensity were multiplied. Tumor specimens were classified into 3 groups according to overall scoring: negative expression as 0 to 1, weak expression as 2 to 4, and high expression as 6 to 12 points. Total scores were as follows: 0 to 4 (low) and 6 to 12 (high). All slides were evaluated independently by 2 pathologists without knowledge of the identity of patients and the clinical outcome.
