# Supplementary figures and images for: Targeting the androgen receptor to enhance NK cell killing efficacy in bladder cancer by modulating ADAR2/circ_0001005/PD-L1 signaling
Source: Cancer Gene Ther. 2022 Aug 1;29(12):1988–2000. doi: 10.1038/s41417-022-00506-w (PMC9750871; doi:10.1038/s41417-022-00506-w)

Supplement Fig.1

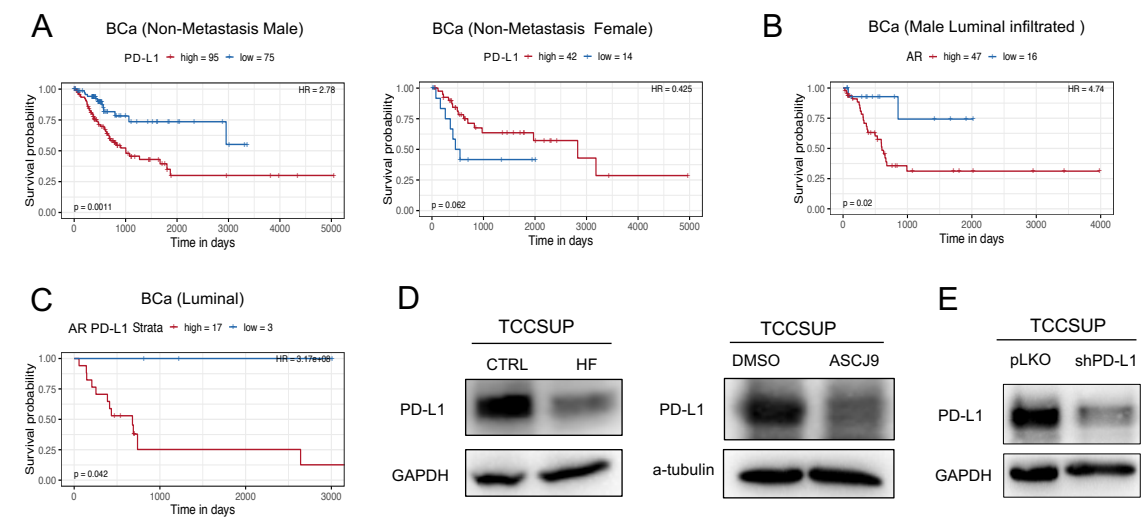

Supplement Fig.2

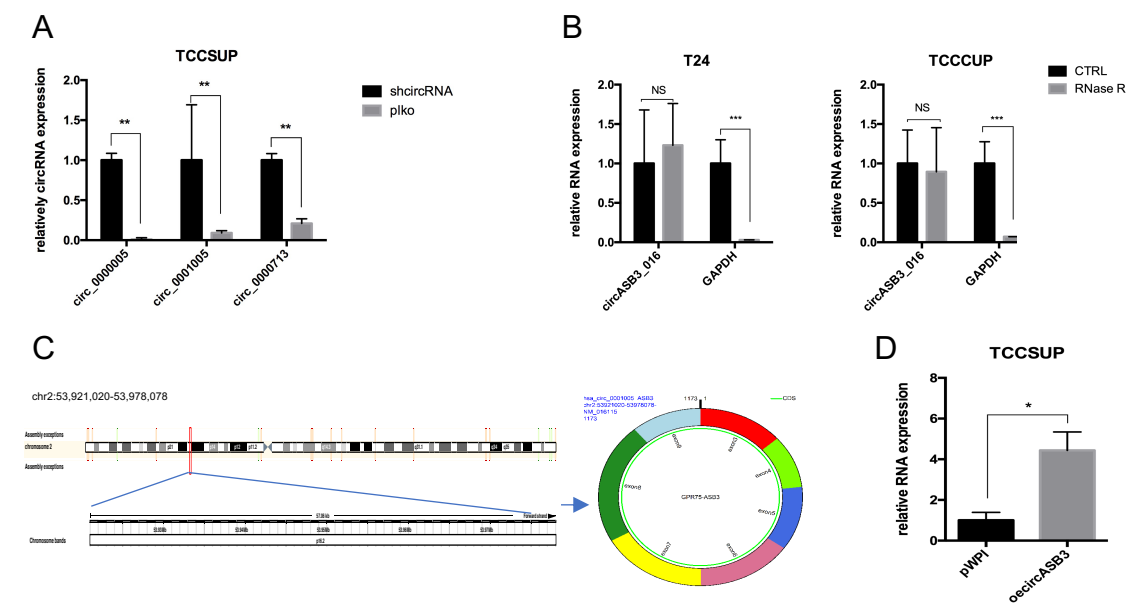

Supplement Fig.3

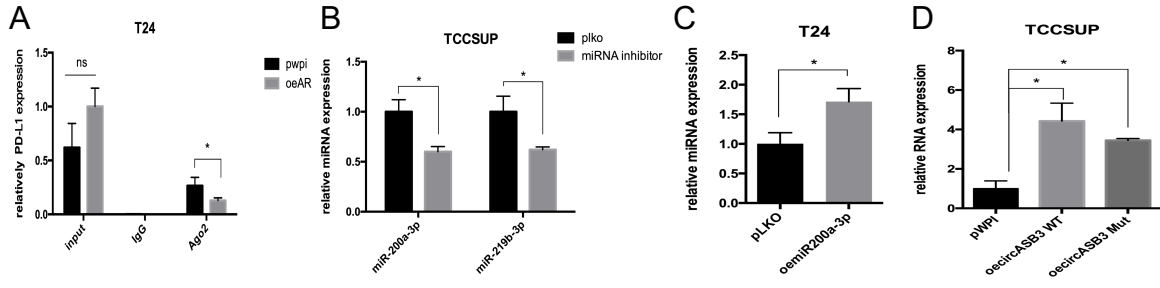

Supplement: Supplementary file 3 — Supplementary figure [file 41417_2022_506_MOESM3_ESM.pdf]
