# Supplementary material for: Targeting the androgen receptor to enhance NK cell killing efficacy in bladder cancer by modulating ADAR2/circ_0001005/PD-L1 signaling
Source: Cancer Gene Ther. 2022 Aug 1;29(12):1988–2000. doi: 10.1038/s41417-022-00506-w (PMC9750871; doi:10.1038/s41417-022-00506-w)
Supplement: Supplementary file 4 — Supplementary table [file 41417_2022_506_MOESM4_ESM.pdf]

Supplemental Table 1. Primers used

ID	Forward primer	Reverse primer
hsa_circ_0000712	5‘-CCAGCGGTCTGCTCAAGAAC-3’	5‘-GAAGGTGCAGAAAGTCGTCCA-3’
hsa_circ_0000005	5’-TTTGGCCATCAAACCACCCC-3’	5’-CGCTTGGAATCCCGGTCATC-3’
hsa_circ_0001005	5’-TTGCATCTCGCTGCAAGTCA-3’	5’-TGCACAGTAGAAATGGTGTCGT-3’
hsa_circ_0001427	5’-TTGGGACAAACCCAGCCAAG-3’	5’-TTGGCATCGACCACACATCG-3’
hsa_circ_0000713	5’-TGATTTGCTTTTCTGCACCTTCA-3’	5’-CACAGTTGCCCCATCAGGTG-3’
hsa_circ_0000780	5’-CACACCAGCAAGTTCAGAATGT-3’	5’-GACCCAGCTGTGCTTTGGAA-3’
hsa_circ_0000249	5’-TCCTACTGTTTGTTGGTGGCA-3’	5’-TTCAGAAGCATGGTGAGCCA-3’
hsa_circ_0001875	5’-TTTCCATGGCTTGGTTGCGT-3’	5’-AGTCAGAGGTGTCCTGTTCCG-3’
hsa_circ_0000006	5’-CTACAACCAGGACGTGGTGC-3’	5’-GTATTTGTCCCCGCTGAGCA-3’
circ_CD274intron	5’-ATTGAACATCTTTCATATGTTTA-3’	5’-TTAGGGGGCACTTTGATACTTCC-3’
hsa-miR-34b-5p	TAGGCAGTGTCATTAGCTGATTG	
hsa-miR-661	TGCCTGGGTCTCTGGCCTGCGCGT	
hsa-miR-32-5p	TATTGCACATTACTAAGTTGCA	
hsa-miR-3160-5p	GGCTTTCTAGTCTCAGCTCTCC	
hsa-miR-6838-5p	AAGCAGCAGTGGCAAGACTCCT	
hsa-miR-373-5p	ACTCAAAATGGGGGCGCTTTCC	
hsa-miR-200a-3p	TAACACTGTCTGGTAACGATGT	
hsa-miR-4448	GGCTCCTTGGTCTAGGGGTA	
hsa-miR-1304-5p	TTTGAGGCTACAGTGAGATGTG	

hsa-miR-3137	TCTGTAGCCTGGGAGCAATGGGGT	
hsa-miR-424-5p	CAGCAGCAATTCATGTTTTGAA	
hsa-miR-221-5p	ACCTGGCATACAATGTAGATTT	
hsa-miR-3133	TAAAGAACTCTTAAAACCCAAT	
hsa-miR-219b-3p	AGAATTGCGTTTGGACAATCAGT	
